# ‘The Virtual Check-In’: A tool to facilitate virtual patient interaction for early clinical learners in a longitudinal integrated clerkship

**DOI:** 10.15694/mep.2020.000108.1

**Published:** 2020-05-22

**Authors:** Aaron Johnston, Kendra Barrick, Farah Jivraj, Rithesh Ram

**Affiliations:** 1 University of Calgary

**Keywords:** Virtual Medicine, Rural Medicine, Longitudinal integrated clerkships, Medical education, Patient encounter tools, Clerkship, Undergraduate Medical Education, Educational Tools

## Abstract

This article was migrated. The article was marked as recommended.

In response to restrictions on learner placements in clinical environments during the COVID-19 pandemic the authors developed a tool, ‘The Virtual Check-In’, for clinical clerks in the University of Calgary Longitudinal Integrated Clerkship. These learners, who had been pulled from their rural and remote communities because of the pandemic, used the tool to continue to develop their clinical skills while working with their preceptors and patients online. This paper describes the rapid development of the tool using Kern’s principles for curriculum development and implementation, the format and uses of the final tool, and its potential use in other contexts.

## Introduction

The University of Calgary Longitudinal Integrated Clerkship (UCLIC) is a clerkship stream that places clinical clerks in in a rural environment for the majority of the clerkship. The UCLIC experience is designed to increase student exposure to continuity of care, rural medicine and generalist principles. Each year approximately 25 students from the class of approximately 150 students choose the UCLIC program. The University of Calgary is an intensive 3-year medical school and UCLIC is the only longitudinal integrated clerkship program that occurs in the terminal year of training, graduating students directly into residency programs ( Myhre *et al.,* 2015). The UCLIC program has been in place since 2008 and has demonstrated equivalent academic outcomes to the traditional block-based clerkship ( Myhre *et al.,* 2014).

Responding to the COVID-19 pandemic all clinical clerks at the University of Calgary were removed from patient care activities in March of 2020, which coincided closely with the starting point of the UCLIC. While students were restricted from direct patient contact many preceptors were also required to move to virtual forms of patient care. The educational value of involving learners in virtual care has been previously described ( Knight *et al.,* 2016), however rapidly implementing teaching within the context of virtual care represented a challenge both for preceptors (who were new to the technology) and for students (who were new to patient care).

Wanting to maintain the value of involving the UCLIC students with medical practices in their assigned communities as much as possible we decided to develop ‘The Virtual Check In’ tool ( Table 1) as a tool to maintain the involvement of early clinical learners in virtual care during this time. We describe the development of the tool and its implementation as a way of helping other clerkships and equivalent programs facing similar challenges.

## Methods

We used a case study methodology ( Yin, 2003) to study the development, implementation and use of the tool. In the initial development of the tool feedback from rural preceptors, UCLIC leadership and UME leadership was solicited and the content and flow of the tool modified, and additional supports for preceptors developed. During the training phase both observation and feedback were used to further refine the tool. Following a period of use, of the tool the investigators plan to again solicit feedback from students and preceptors to allow further refinement of the tool. We describe both the tool itself as well as the lessons learned from development and implementation.

## Design Principles

In developing our tool, we relied on three guiding principles; i) It must be student-centred and meet institutional educational requirements, ii) it must be easy and intuitive for busy preceptors to implement, and iii) it must be safe for patients. We used Kern’s six step approach to curriculum development as the theoretical framework for development ( Thomas *et al.,* 2015) and describe our process to date below using Kern’s six steps.

1. Problem identification and general needs assessment: Clinical clerks are restricted from in-person clinical encounters and are located at a distance to their LIC communities. Preceptors are engaged in new clinical environments and new virtual tools in response to COVID-19 leaving limited time to learn new supervision techniques and to possibly less time to check in with patients who are not acutely ill.
2. Targeted needs assessment: Clinical clerks need opportunities to continue to build and refine clinical skills. Preceptors need specific support implementing virtual technology and transitioning existing teaching skills to a virtual environment.
3. Goals and measurable objectives: Develop a tool that allows an early clinical learner to conduct a virtual encounter with a patient, gather information and report information to a preceptor. Enable LIC preceptors to continue to effectively teach clinical clerks in a virtual environment as evidenced by rate of participation in the program, satisfaction with the program and feedback on usefulness of the tool.
4. Educational strategies: Provide standardized training to students using the tool. Provide preceptors resources and direct support in teaching and virtual care.
5. Implementation: To prepare students to use the tool a simulation case was prepared by one of the rural preceptors and run with students and standardized patients. Observation and debriefing were carried out by rural LIC preceptors. To prepare preceptors for teaching using virtual care a webinar was created and had over 100 live participants ( Johnston *et al.,* 2020). The webinar was also recorded, and preceptors were provided with the link for reference. Preceptors also received copies of materials supporting teaching in virtual care provided by the College of Family Physicians of Canada ( College of Family Physicians of Canada, 2020, Oandasan *et al.,* 2020).
6. Evaluation and feedback:The tool will be evaluated through student and preceptor surveys. The rate of student and preceptor participation will be measured. Surveys will include quantitative information about how frequently the tool was used and ratings of usefulness as well as qualitative feedback about the utility of the tool from both preceptor and student perspectives.

After the initial draft of the tool was created, we sought feedback from learners and rural preceptors as partners in the development process. At the implementation stage rural preceptors were involved in creating and supervising a simulation using the tool. We utilized standardized patients in the simulation to allow clinical clerks to first use the tool in a safe and controlled environment. Following the simulation we again sought feedback from both students and rural preceptors and again revised the tool.

## The Virtual Check-In Tool

Our Virtual Check-In tool ( Table 1) was designed for early clinical learners to guide virtual interactions with their patients. The structure of the tool was designed to facilitate a virtual interaction with a patient allowing structured information gathering and transmission of information back to the preceptor. The check in is different than many typical patient visits because it is not issue driven. Instead it is designed to gather information that can identify issues that may need to be subsequently addressed in future encounters. Information is recorded for each aspect of the encounter and communicated with the supervising preceptor. Learners and preceptors are required to have a set communication strategy to immediately involve the preceptor in an encounter should acute or serious concerns arise.

**Table 1.** The Virtual Check-In tool

	Information
**Preparation**	*Virtual visit etiquette:* *Consider the space that the patient will view during a virtual visit.* *Consider sign-posting your actions to the patient if you are not looking at them. E.g. typing up notes on the EMR.* *Sending documents:* *If working away from the clinic office, consider how to transfer documents E.g. prescriptions, lab and imaging requisitions. Could an MOA send this from the clinic? Consider testing the EMR from home to see what is possible.* *Does the patient need access to a printer?* *Follow-up visit:* *Consider the workflow for arranging a follow-up visit for the patient. What are the instructions for the patient?* *If patient does not answer, note the time, date, phone number, and message provided if one was left on the progress note*
**Introduction**	*Introduce yourself and the nature of the encounter. Confirm the patient’s identity. Acknowledge the unique situation by thanking the patient for speaking with you virtually. Set time expectation for the encounter.*
**Consent to virtual interaction**	*Ensure patient understands and verbally consents to the virtual interaction. Use scripted consent statement if using non-secured technology as suggested by the College of Physicians and Surgeons of Alberta.* *“Unregulated virtual care technologies increase the risk that your personal health information may be intercepted or disclosed to third parties. These tools are being used as an extraordinary measure during the COVID-19 pandemic when regulated technology is not readily available, and the necessity to keep people from congregating or attending health facilities where they may be exposed to the COVID-19 virus is thought to outweigh the risk of personal privacy breaches on both a personal and population health basis.” ( College of Physicians and Surgeons of Alberta, 2020)*
**Basic needs assessment**	*Are the patient’s basic needs for housing, food, companionship, etc. currently being met?*
**Wellness and mental health**	*Inquiry into how the patient is feeling. Encourage* *and support general wellness measures such as exercise, healthy eating, maintaining structured routine, hobbies/interest and virtual social engagement*
**Medications**	*Does patient have adequate supply of current medications based on what pharmacies are able to dispense at one time? Are there any concerns with any medications (e.g.: side effects, compliance, not working, etc.)*
**Medical concerns**	*Does patient have any new medical concerns that they need their physician to be aware of that they would like to have addressed or reviewed?*
**Other**	*Other important issues that emerge during the encounter.*
**Conclusion and follow-up**	*Remind patient that information will be communicated to the preceptor, and a follow-up encounter may occur. Provide the patient with the opportunity to state that they would definitely like to speak with the family physician.*

## Discussion

The Virtual Check-In tool has allowed final year medical students enrolled in the UCLIC program to continue to connect with patients in their assigned communities during the COVID pandemic. This in turn supports continuity of care, a core principle of the UCLIC program. Students working with a single medical practice during UCLIC have in this way been afforded the opportunity to check in with the same patients across time and to see those same patients in person when normal clinical activities resume.

The tool has followed the principles outlined in the development phase; meeting students educational needs by allowing student patient interaction, being simple for busy preceptor to implement, and maintaining structured supervision to ensure patient safety.

A variety of tools and resources supporting virtual care exist, but we were unable to find any published tools specifically designed to support virtual care for early clinical learners. High quality resources such as the Virtual Care Playbook ( Dermer, 2020) guide physicians on both the technical and logistic aspects of virtual care and are aimed at the level of the practicing physician. Our tool is aimed at the early clinical learner specifically and is much more structured than a typical patient-physician encounter.

Due to the rapid development of the COVID pandemic and its systemic disruption to everyday educational programing, the development and implementation of our tool occurred over a much faster timeline than is typical. This limited our ability to use formal research methods to inform and study the development of the tool.

In order to implement this tool in other environments the tool would likely require local review and potentially some form of translation to ensure that local professional and legal norms were adhered to, in particular regulations around virtual care, privacy and consent. Alignment with the local curriculum expectations and requirements would also be necessary. Finally, given that the tool also includes inquiries into potentially sensitive topics, basic needs, mental health and general health issues the tool may require modification to align with different cultural contexts. Nevertheless, we commend the tool and the approach to the medical education community as a way of maintaining educational experiences for distributed medical education learners despite disruptions to their face-to-face learning.

Future work will include evaluation and validation testing with patients, preceptors, and learners and iterative refinements based on these evaluations of the tool in use. We are also interested in exploring whether (and if so when and how) the tool may continue to be used once the program returns to on-site learning for UCLIC students.

## Conclusion

Using Kern’s framework to guide the rapid development and deployment of an educational tool we were able to allow our LIC students to participate in virtual care in response to the COVID-19 pandemic. We believe that the tool will allow students to continue to develop clinical skills and to subsequently meet educational requirements, that the tool will be safe and beneficial for patients and straightforward for preceptors to implement. We hope that sharing our tool as well as our development and implementation process as it is likely other LICs and clerkships are facing similar challenges and may benefit from access to this tool.

## Take Home Messages

• The Virtual Check-In tool allows early clinical learners (clinical clerks), restricted from clinical environments due to COVID, to continue patient centred learning through virtual care.
• The Virtual Check-In tool is easy for both learners (clinical clerks) and preceptors to use and standardizes supervision to ensure safety of patients.
• Virtual care can allow learners in rurally based Longitudinal Integrated Clerkships to continue interacting with patients and preceptors in their rural communities while restricted from clinical learning environments due to COVID.
• Rapid development of educational tools to meet immediate educational challenges can be accomplished using the Kern framework for curriculum development.

## Notes On Contributors

**Dr. Aaron Johnston** M.D. is the Associate Dean of Distributed Learning and Rural Initiatives and a Clinical Associate Professor in the Departments of Emergency Medicine and Family Medicine at the University of Calgary. He is also the Chair-elect of the Section of Teachers of the College of Family Physicians of Canada.

**Dr. Kendra Barrick** M.D. is a Primary Preceptor in the University of Calgary Longitudinal Integrated Clerkship and a Clinical Lecturer in the Department of Family Medicine at the University of Calgary.

**Dr. Farah Jivraj** MB BCh BAO is the Assistant Director of the University of Calgary Longitudinal Integrated Clerkship and a Clinical Lecturer in the Department of Family Medicine at the University of Calgary.

**Dr. Rithesh Ram** M.D. Ph.D. (Epidemiology) is the Director of the University of Calgary Longitudinal Integrated Clerkship and a Clinical Assistant Professor in the Department of Family Medicine at the University of Calgary.
