# Supplementary material for: ‘The Virtual Check-In’: A tool to facilitate virtual patient interaction for early clinical learners in a longitudinal integrated clerkship
Source: MedEdPublish (2016). 2020 May 22;9:108. [Version 1] doi: 10.15694/mep.2020.000108.1 (PMC10702631; doi:10.15694/mep.2020.000108.1)
Supplement: Peer review history

# Reviewer report: Reviewer response for version 1

**DOI:** 10.21956/mep.20084.r30921

**Published:** 2020-10-15

**Authors:** Ken Masters

**Affiliations:** 1 Sultan Qaboos University

This review has been migrated. The reviewer awarded 3 stars out of 5 An interesting paper dealing with a tool to facilitate virtual patient interaction for early clinical learners in a longitudinal integrated clerkship. The authors describe the overall problem brought on by COVID-19, and the need to protect their students and patients, and yet still ensure that their students receive the required training. Using an established curriculum development framework, they developed their tool and implemented it.Overall, this is a useful paper, but I would like to see some expansion:• Although the tool has appeared to be successful, I would urge the author to look at the complexity of the scripted consent - it is rather a torrent of words. While I am not suggesting that this be changed (I presume they have limitations), I would like to know how the student (or any clerk) confirmed that the patient understood this. I hope it was not merely a case of “Do you understand?” to be met with the obligatory “Yes”, which would have the same legal impact, but also the same lack of meaning, as checking on the “I have read the terms and conditions” checkbox. (In this context, it would also be useful to have some idea of the language background of the patients currently being served).• The time allowed for the check-in, and how this differed from physical check-ins. I realise that, as this is not a full research article, full details have not necessarily been tracked, but it would be useful for readers to know how much time was allowed, and the time range that was actually experienced.• Again, although this is not a research article, some indication of an evaluation would be useful.I look forward to Version 2 of this paper in which there is some expansion on these issues.

Reviewer Expertise:

NA

No decision status is available

# Reviewer report: Reviewer response for version 1

**DOI:** 10.21956/mep.20084.r30922

**Published:** 2020-05-31

**Authors:** Shazia Iqbal

**Affiliations:** 1 Alfarabi College of Medicine Riyadh, KSA

This review has been migrated. The reviewer awarded 4 stars out of 5 Sending documents:If working away from the clinic office, consider how to transfer documents E.g. prescriptions, lab and imaging requisitions.

Reviewer Expertise:

NA

No decision status is available

# Reviewer report: Reviewer response for version 1

**DOI:** 10.21956/mep.20084.r30919

**Published:** 2020-05-26

**Authors:** Alexandra Webb

**Affiliations:** 1 Australian National University

This review has been migrated. The reviewer awarded 3 stars out of 5 This is an interesting article describing the development of a virtual check-in tool designed to facilitate the continuation of clinical learning opportunities for students when these were not possible during the covid crisis. For readers not familiar with the medical training system in Canada it would be helpful to define terms such as preceptor and clinical clerk. The details regarding the Check-In Tool will be helpful to medical educators seeking a similar solution when clinical placements are not possible. The addition of information regarding the affordances of the technology utilised for delivering the Check-In Tool would be helpful e.g. audio-only, audio and video.

Reviewer Expertise:

NA

No decision status is available

# Reviewer report: Reviewer response for version 1

**DOI:** 10.21956/mep.20084.r30920

**Published:** 2020-05-26

**Authors:** P Ravi Shankar

**Affiliations:** 1 American International Medical University

This review has been migrated. The reviewer awarded 4 stars out of 5 I read with the interest the article about the development of an online check-in tool for use during longitudinal integrated clerkships at the University of Calgary. The authors have developed and described the process very well. Some more information about the intensive three-year MD program at the university and the place of the longitudinal clerkship within it will be of interest. Table 1 describes the tool very well. I found some of the steps during the preparation phase a bit difficult to understand. The tool can be modified and adapted for use in other settings as mentioned by the authors.

Reviewer Expertise:

NA

No decision status is available
